# Inhibition of Mitochondrial Metabolism Leads to Selective Eradication of Cells Adapted to Acidic Microenvironment

**DOI:** 10.3390/ijms221910790

**Published:** 2021-10-06

**Authors:** Martina Koncošová, Nikola Vrzáčková, Ivana Křížová, Petra Tomášová, Silvie Rimpelová, Aleš Dvořák, Libor Vítek, Michaela Rumlová, Tomáš Ruml, Jaroslav Zelenka

**Affiliations:** 1 Department of Biochemistry and Microbiology, University of Chemistry and Technology Prague, 166 28 Prague, Czech Republic; martina.koncosova@vscht.cz (M.K.); nikola.vrzackova@vscht.cz (N.V.); silvie.rimpelova@vscht.cz (S.R.); tomas.ruml@vscht.cz (T.R.); 2 Department of Biotechnology, University of Chemistry and Technology Prague, 166 28 Prague, Czech Republic; ivana.krizova@vscht.cz (I.K.); michaela.rumlova@vscht.cz (M.R.); 3 Institute of Medical Biochemistry and Laboratory Diagnostics, Faculty General Hospital and 1st Faculty of Medicine, Charles University, 120 00 Prague, Czech Republic; petra.lacinova@lf1.cuni.cz (P.T.); ales.dvorak@lf1.cuni.cz (A.D.); libor.vitek@lf1.cuni.cz (L.V.); 4 Institute of Microbiology, The Czech Academy of Sciences, 140 00 Prague, Czech Republic; 5 4th Department of Internal Medicine, Faculty General Hospital and 1st Faculty of Medicine, Charles University, 120 00 Prague, Czech Republic

**Keywords:** tumor microenvironment, mitochondria, acidosis, photodynamic therapy, CPI-613, tetracycline, lactate, cancer, bioenergetics, therapy

## Abstract

Metabolic transformation of cancer cells leads to the accumulation of lactate and significant acidification in the tumor microenvironment. Both lactate and acidosis have a well-documented impact on cancer progression and negative patient prognosis. Here, we report that cancer cells adapted to acidosis are significantly more sensitive to oxidative damage induced by hydrogen peroxide, high-dose ascorbate, and photodynamic therapy. Higher lactate concentrations abrogate the sensitization. Mechanistically, acidosis leads to a drop in antioxidant capacity caused by a compromised supply of nicotinamide adenine dinucleotide phosphate (NADPH) derived from glucose metabolism. However, lactate metabolism in the Krebs cycle restores NADPH supply and antioxidant capacity. CPI-613 (devimistat), an anticancer drug candidate, selectively eradicates the cells adapted to acidosis through inhibition of the Krebs cycle and induction of oxidative stress while completely abrogating the protective effect of lactate. Simultaneous cell treatment with tetracycline, an inhibitor of the mitochondrial proteosynthesis, further enhances the cytotoxic effect of CPI-613 under acidosis and in tumor spheroids. While there have been numerous attempts to treat cancer by neutralizing the pH of the tumor microenvironment, we alternatively suggest considering tumor acidosis as the Achilles’ heel of cancer as it enables selective therapeutic induction of lethal oxidative stress.

## 1. Introduction

The metabolic transformation has been recently included between the hallmarks of cancer cells. Its principal component, the Warburg effect, is characterized by the enhanced utilization of glucose and the excessive production of lactate even in the presence of oxygen and a competent mitochondrial respiratory chain [1]. The resulting accumulation of lactate is accompanied by the acidification of the tumor microenvironment which is further pronounced by tumor hypercapnia and the activity of carbonic anhydrases IX and XII (CAIX, CAXII) intended to ameliorate intracellular acidosis on account of the extracellular pH [2].

The presence of lactic acidosis selects a subpopulation of oxidative cancer cells with enhanced activity of mitochondrial respiratory chain and malignant potential [3,4,5]. Both lactate content and acidity of the tumor microenvironment have been associated with the occurrence of metastases and poor patient prognosis [6,7,8,9]. Moreover, lactic acidosis plays a well-documented role in tumor neoangiogenesis and the induction of tolerance by infiltrated immune cells [10,11,12]. Therefore, CAIX/CAXII [13,14], lactate dehydrogenase [15,16,17], monocarboxylate transporters responsible for the import/export of lactate and H^+^ through the cytoplasmic membrane [17,18,19], and other components involved in the formation of the lactic acidosis have been pharmacologically targeted in an attempt to block the cancer progression. However, none of these approaches achieved the phase III clinical trial.

On the other hand, cancer cells are also characterized by enhanced production of reactive oxygen species (ROS) triggered by the combination of metabolic transformation, oncogenic signaling, and eventually mutations in metabolic genes including mitochondrial DNA (deoxyribonucleic acid) [20]. The compensation of resulting oxidative stress with a strong antioxidant response is critical for cell survival and cancer progression [21]. Moreover, high antioxidative capacity stimulates tumor metastasis [22,23] and cancer resistance to therapy [24,25].

Interestingly, both the elevation of extracellular lactate [26] and acidosis [27,28] have been independently demonstrated to enhance ROS production and antioxidant response. A few works investigating the effect of lactic acidosis on cell viability and resistance to oxidative stress pointed out the importance of genetic background and the difference between simple acidosis and lactic acidosis [28,29]. This study aimed to separately identify the effects of lactate and acidosis on the sensitivity of cancer cells to oxidative stress-based anticancer therapies.

## 2. Results and Discussion 

### 2.1. The Effects of Lactic Acidosis on the Cell Sensitivity to Oxidative Insult

**Figure 1:**
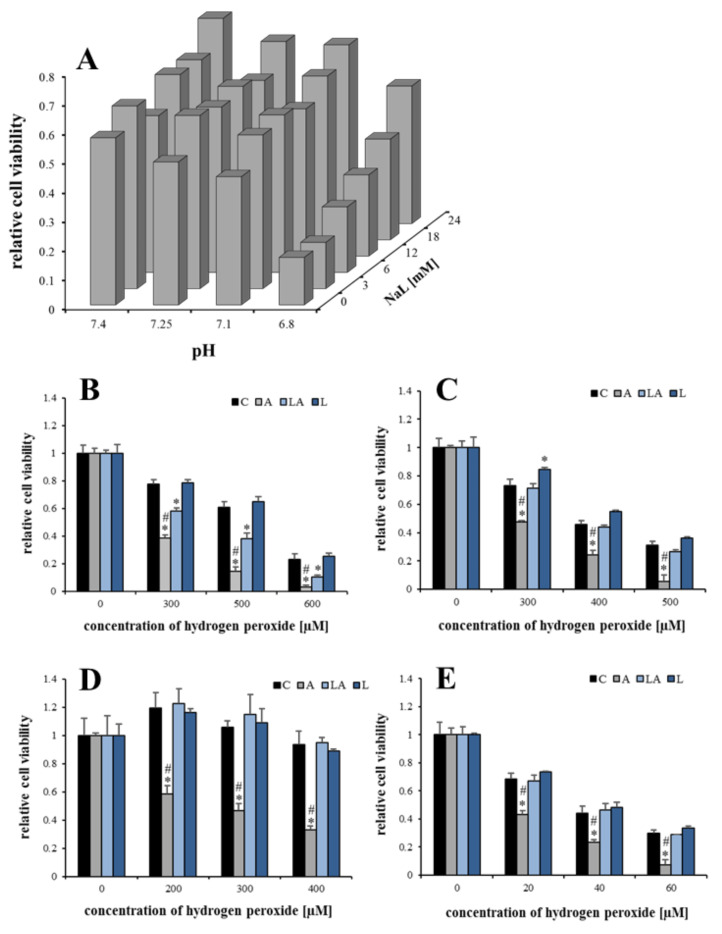
The effect of lactic acidosis on cell sensitivity to oxidative insult. (**A**) Viability of PaTu-8902 cells cultured in media of different pH and concentrations of lactate anion (NaL) for 72 h and then treated with 500 µM hydrogen peroxide. Viability of (**B**) PaTu-8902, (**C**) HeLa, (**D**) Hep G2, and (**E**) noncancerous HDF cells cultured in experimental media for 72 h and then treated with indicated concentrations of hydrogen peroxide. Viability was measured by resazurin assay. C, control; A, acidosis; LA, lactic acidosis; L, high lactate (* *p* < 0.05 compared to C, # *p* < 0.05 when comparing A and LA).

**Figure A1:**
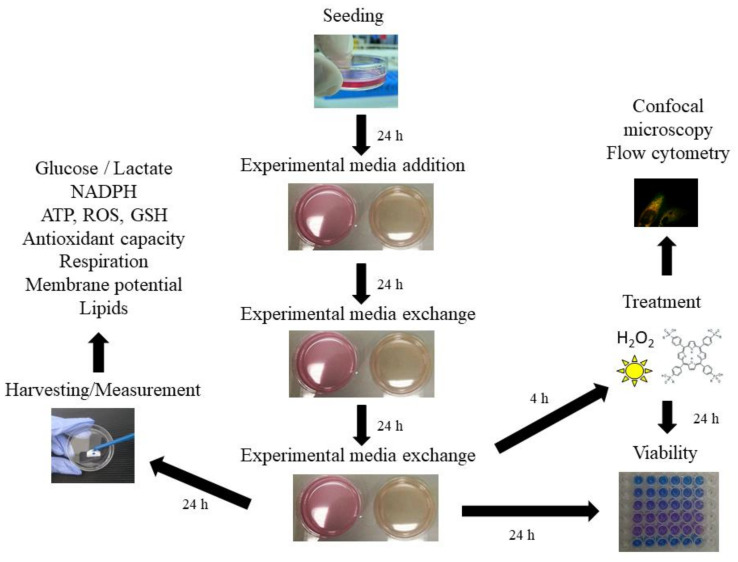
The summary of the cell treatment with experimental media and subsequent measurements.

**Figure A2:**
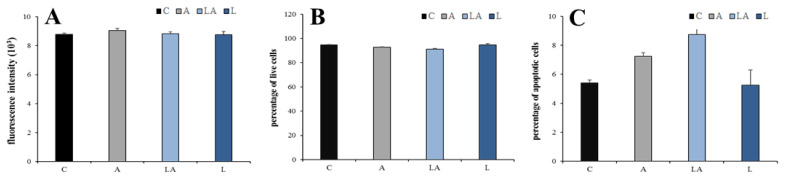
The effect of lactic acidosis on cancer cell viability. Viability (**A**) of PaTu-8902 cells cultured in experimental media for 72 h. Percentage of living (**B**) and apoptotic (**C**) cells in the cell population cultured in experimental media for 72 h. Viability was measured by resazurin assay. Apoptosis was determined using flow cytometry. C, control; A, acidosis; LA, lactic acidosis; L, high lactate.

**Figure A3:**
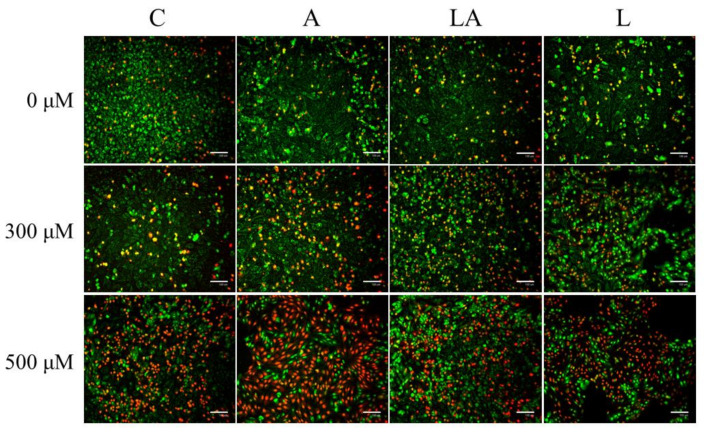
Live/dead staining of the cells treated with hydrogen peroxide. HeLa cells were cultured in experimental media for 72 h and treated with indicated concentrations of hydrogen peroxide. Total cells were stained with SYTO-9 (green), dead cells were stained with propidium iodide (red). C, control; A, acidosis; LA, lactic acidosis; L, high lactate.

Lactate and acidosis were linked to ROS burst and upregulation of antioxidant defense [26,27,28,30,31,32]. However, little is known about the individual effects of lactate and acidosis on cancer cell resistance to lethal oxidative stress. Therefore, in this study, human pancreatic PaTu-8902 cells were treated with cell culture media with different concentrations of the lactate anion and pH, 0–24 mM and 6.8–7.4, respectively, for three days to enable metabolic adaptation. To approximate the physiological conditions of the human body, the medium with 5 mM glucose and 0.5 mM glutamine, which was exchanged daily, was employed. The whole experimental design is summarized in Figure A1 in Appendix A. The treatment with the experimental media resulted in only marginal changes in cell viability and the occurrence of apoptotic cells (Figure A2). Cells were subsequently exposed to 500 µM hydrogen peroxide as an oxidative insult for 24 h. There was a significant dose-dependent relationship among pH, lactate levels, and the observed decrease in cell viability. While acidosis significantly compromised cell viability, the presence of lactate anion has a protective effect. Importantly, the presence of lactate in acidic media significantly and dose-dependently mitigated the effect of acidosis on the cell sensitivity to oxidative stress (Figure 1A). This phenomenon was detected at a pH of 6.8 and a 12 mM concentration of lactate, i.e., the conditions previously reported for the tumor microenvironment [4,33,34]. Therefore, the general validity of this phenomenon was demonstrated under these conditions using three different human cancer cell lines (PaTu-8902, cervix HeLa, and liver Hep G2), a noncancerous human dermal fibroblast (HDF) cell line, and several concentrations of hydrogen peroxide (Figure 1B–D). Although some variability regarding the magnitude of the effects and the overall sensitivity of the cell lines to hydrogen peroxide was observed, the sensitization under acidic conditions and its attenuation with lactate was confirmed in all cell lines including the noncancerous one. Live/dead staining correlated with the viability results, revealing a dramatic rise in the population of dead cells with increasing concentration of hydrogen peroxide (Figure A3).

**Figure 2:**
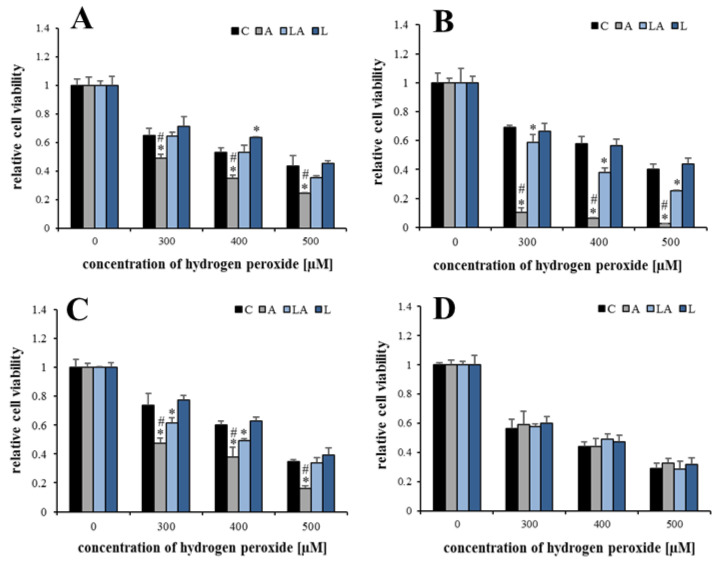
The effect of adaptation time on cell sensitivity to oxidative insult. PaTu-8902 cells were treated with experimental media for (**A**) 6 h, (**B**) 14 days, and (**C**,**D**) 72 h followed by 4 and 48 h washout with the control medium, respectively. Then, the cells were treated with the indicated concentrations of hydrogen peroxide and their viability was measured by resazurin assay. C, control; A, acidosis; LA, lactic acidosis; L, high lactate (* *p* < 0.05 compared to C, # *p* < 0.05 when comparing A and LA).

To establish whether these effects are directly caused by harmful extracellular conditions or whether they resulted from cell adaptation, PaTu-8902 cells were treated with the experimental media for various lengths of time before the treatment with hydrogen peroxide. The effects were observed already 6 h post-treatment with the experimental media (Figure 2A), although with a smaller magnitude than after 72 h (Figure 1B). In contrast, the treatment lasting 14 days further intensified the effects (Figure 2B) suggesting that the effects did not vanish during the long-term adaptation. When the cells were treated with the experimental media for 72 h and then with the control medium for 4 h, the effect was still present, although with a smaller magnitude (Figure 2C). Thus, the effects resulted from cell adaptation to lactic acidosis rather than from the harmful effects of acidosis itself. Finally, the effects disappeared completely after 48 h from exchanging the experimental media for the control one (Figure 2D).

### 2.2. The Effect of Lactic Acidosis on Cancer Cell Sensitivity to Therapy

**Figure 3:**
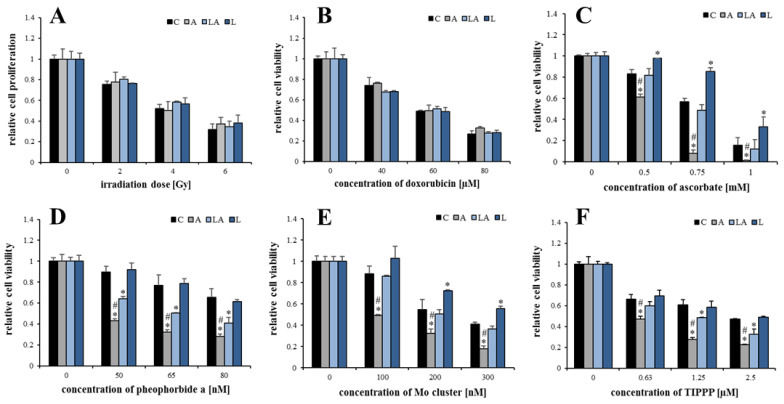
The effect of lactic acidosis on cancer cell sensitivity to radio-, chemo-, and phototherapy. PaTu-8902 cells were cultured in experimental media for 72 h and then treated with (**A**) X-ray irradiation, (**B**) doxorubicin, (**C**) pharmacological ascorbate, and (**D**–**F**) photodynamic therapy using pheophorbide *a*, Mo cluster, and 5,10,15,20-tetrakis(4-isopropylphosphinatophenyl)porphyrin (TIPPP), respectively. The proliferation of irradiated cells after reseeding and subsequent 72 h incubation and viability of the rest after 24 h were measured by resazurin assay. C, control; A, acidosis; LA, lactic acidosis; L, high lactate (* *p* < 0.05 compared to C, # *p* < 0.05 when comparing A and LA).

It has been demonstrated that some cancer therapies are accompanied by oxidative damage [20,21]. Therefore, the effects of lactate and acidosis on the cancer cell sensitivity to such therapies were investigated. PaTu-8902 cells treated with the experimental media for 72 h were exposed to X-ray irradiation (Figure 3A), doxorubicin (Adriamycin, Figure 3B), cytotoxic doses of ascorbate (vitamin C, Figure 3C), and photodynamic therapy (PDT) with three different photosensitizers pheophorbide *a* (Figure 3D), molybdenum (Mo) cluster (Figure 3E), and porphyrin abbreviated as TIPPP (5,10,15,20-tetrakis(4-isopropylphosphinatophenyl)porphyrin, Figure 3F), previously characterized by our group [28,29,30].

**Figure A4:**
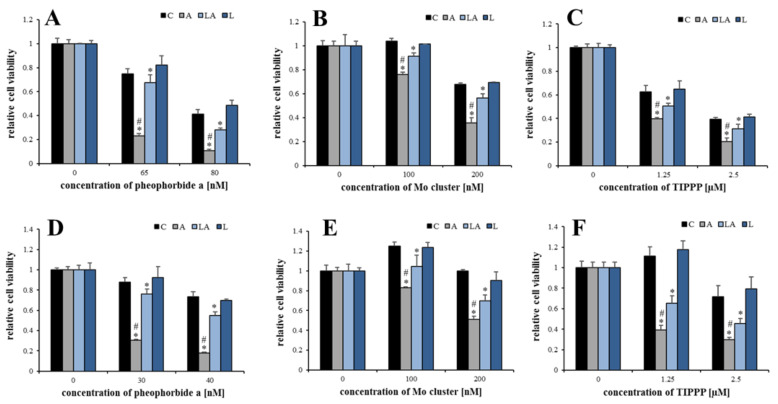
The effect of lactic acidosis on cancer cell sensitivity to photodynamic therapy. Viability of HeLa (**A–C**) and Hep G2 (**D–F**) cells cultured in experimental media for 72 h and then treated with photodynamic therapy using (**A**,**D**) pheophorbide *a*, (**B**,**E**) Mo cluster, and (**C**,**F**) 5,10,15,20-tetrakis(4-isopropylphosphinatophenyl)porphyrin (TIPPP). Viability was measured by resazurin assay. C, control; A, acidosis; LA, lactic acidosis; L, high lactate (* *p* < 0.05 compared to C, # *p* < 0.05 when comparing A and LA).

Interestingly, no effects of the experimental media were observed for X-rays and doxorubicin that act mainly through the DNA damage accompanied by oxidative stress. On the contrary, cells adapted to acidosis were sensitized to pharmacological ascorbate and PDT, which are directly mediated by oxidative damage [35]. The presence of lactate again abrogated the effect of acidosis. The effects of the experimental media on cell sensitivity to PDT were demonstrated also for other cell lines (Figure A4).

### 2.3. The Effect of Lactic Acidosis on Uptake and Localization of Photosensitizers

**Figure 4:**
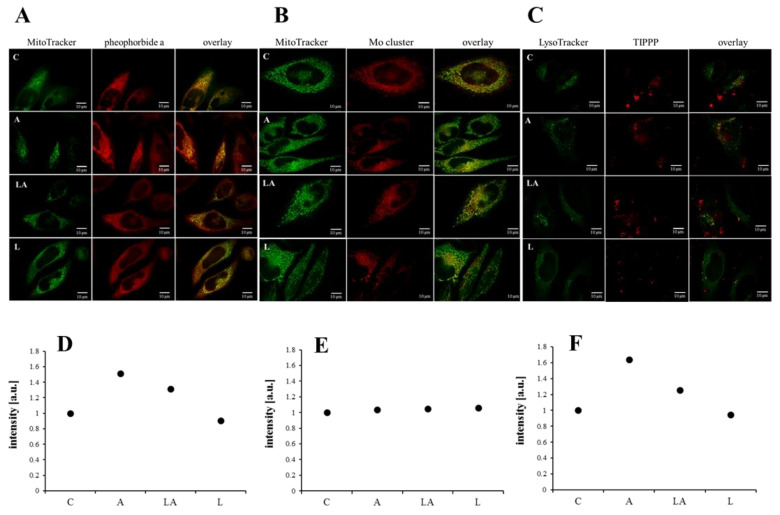
The effect of lactic acidosis on uptake and localization of photosensitizers in cancer cells. Localization of (**A**) pheophorbide *a*, (**B**) Mo cluster, and (**C**) 5,10,15,20-tetrakis(4-isopropylphosphinatophenyl)porphyrin (TIPPP) in HeLa cells treated with the experimental media for 72 h. The photosensitizers (red emission) were co-localized with (**A**,**B**) MitoTracker^TM^ Green FM or (**C**) LysoTracker^TM^ Green DND-26 (both green emission) to visualize the subcellular localization. Uptake of (**D)** pheophorbide *a*, (**E**) Mo cluster, and (**F**) TIPPP in PaTu-8902 cells treated with the experimental media for 72 h measured by flow cytometry. C, control; A, acidosis; LA, lactic acidosis; L, high lactate.

The fluorescence and different physico-chemical properties of the used photosensitizers were utilized for the investigation, whether the observed effects of the experimental media are related to altered uptake or subcellular localization of the compounds used for the induction of oxidative damage. Via live-cell spinning disc confocal microscopy of HeLa cells, we have documented that pheophorbide *a* (Figure 4A) and Mo cluster (Figure 4B) co-localized with mitochondria, while TIPPP co-localized with lysosomes (Figure 4C). No effect of the experimental media on the intracellular localization of the photosensitizers was detected.

**Figure A5:**
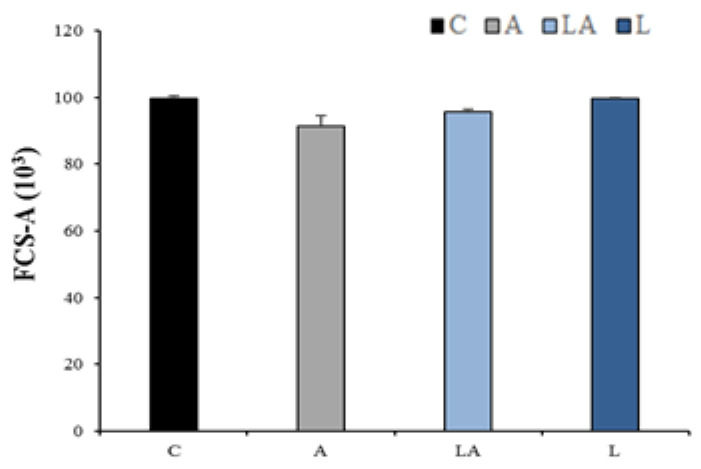
The cell size. Measured as a forward scatter of flow cytometry of PaTu-8902 cells treated with experimental media for 72 h. C, control; A, acidosis; LA, lactic acidosis; L, high lactate.

In addition, the flow cytometry analysis with PaTu cells showed increased uptake of pheophorbide *a* (Figure 4D) and TIPPP (Figure 4F) in the acidic medium. However, interestingly, the penetration of the Mo cluster (Figure 4E) was not affected by the experimental media. Furthermore, there was no effect of experimental media on cell size (Figure A5). Since the changes in the uptake of both porphyrins were insufficient to fully explain the effects of the experimental media, and the cell uptake of the Mo cluster was not affected at all, the observed effects are unlikely to be explained by a differential uptake of the employed compounds. 

### 2.4. The Effect of Lactic Acidosis on Redox Homeostasis of the Cancer Cells

**Figure 5:**
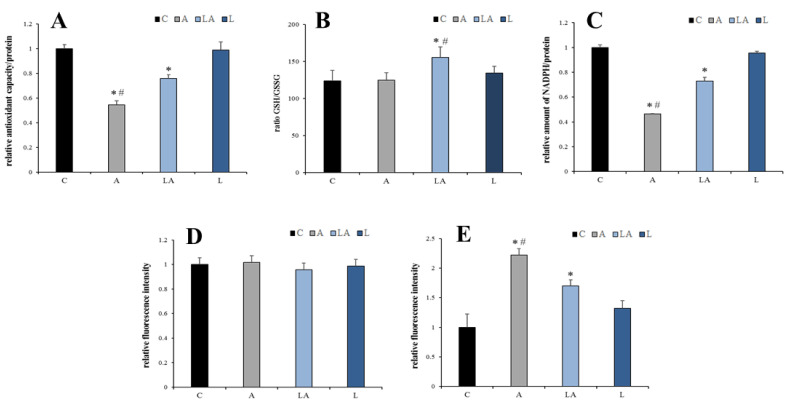
The effect of lactic acidosis on redox homeostasis of cancer cells. PaTu-8902 cells were treated with the experimental media for 72 h and (**A**) relative antioxidant capacity, (**B**) ratio of reduced (GSH) to oxidized (GSSG) glutathione in the cells, (**C**) relative concentration of NADPH, (**D**) oxidation of dihydroethidium, and (**E**) oxidation of 2′,7′-dichlorodihydrofluorescein measured as a relative fluorescence were determined. C, control; A, acidosis; LA, lactic acidosis; L, high lactate (* *p* < 0.05 compared to C, # *p* < 0.05 when comparing A and LA).

Next, the role of cell redox homeostasis in differential sensitivity to oxidative insult was investigated. Determination of the total cellular antioxidant capacity revealed a significantly compromised antioxidant barrier in PaTu-8902 cells treated with the acidic medium (Figure 5A). The simultaneous presence of lactate significantly improved the antioxidant status of the cells. However, the concentration and redox status of glutathione, the essential intracellular antioxidant [21], were not consistently affected by the experimental media (Figure 5B).

Since the redox status of glutathione and other antioxidants is determined by continuous oxidation with ROS and reduction with NADPH [36], these variables were also studied. The levels of total intracellular NADPH were significantly decreased in acidic media, while the simultaneous presence of lactate significantly mitigated this effect (Figure 5C). Production of superoxide, the prime cellular ROS produced by mitochondria and NADPH oxidases [36], measured as oxidation of dihydroethidium probe, was not affected by the experimental media (Figure 5D). In contrast, the oxidation of 2′,7′-dichlorodihydrofluorescein (DCF), was significantly increased under acidosis, which was attenuated by the simultaneous presence of lactate (Figure 5E). The oxidation of DCF is thought to be caused by peroxyl radicals and other dangerous, short-lived products of the oxidative damage resulting from the breakthrough of the antioxidant barrier [37], which seems to be the case under acidic conditions. Therefore, these data suggest that the sensitization of the cells to oxidative insult in the acidic medium is mediated by compromised antioxidant machinery driven by insufficient levels of NADPH.

### 2.5. The Effect of Lactic Acidosis on the Cancer Cell Metabolism

**Figure 6:**
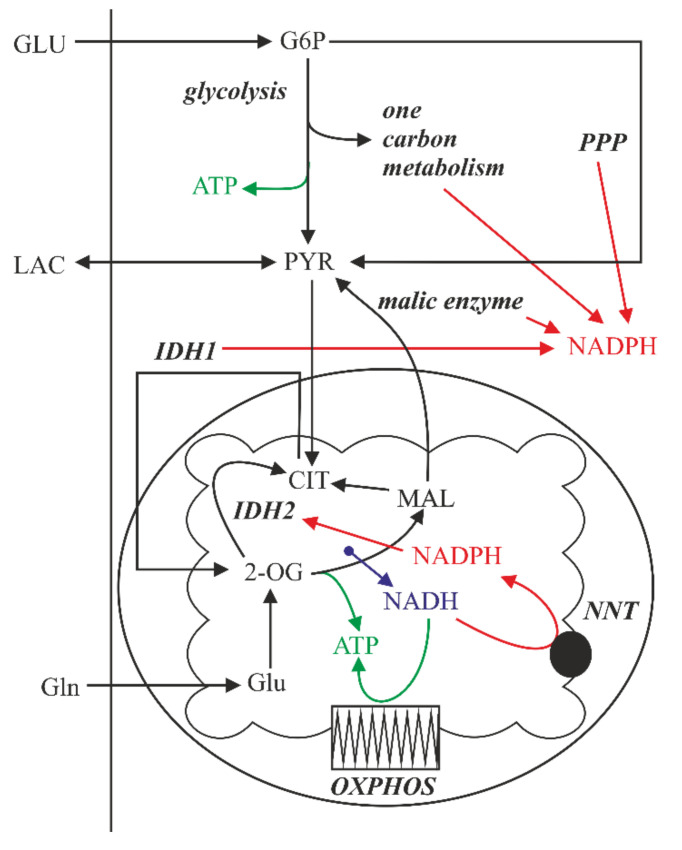
Partitioning of metabolic flow between the production of ATP and NADPH. ATP—adenosine triphosphate, CIT—citrate, GLU—glucose, G6P—glucose-6-phosphate, Gln—glutamine, Glu—glutamate, IDH1(2)—isocitrate dehydrogenase 1(2), LAC—lactate, MAL—malate, NAD(P)H—nicotinamide adenine dinucleotide (phosphate), NNT—nicotinamide nucleotide transhydrogenase, 2-OG—2-oxoglutarate, OXPHOS—mitochondrial oxidative phosphorylation system, PPP—pentose phosphate pathway, PYR—pyruvate.

**Figure 7:**
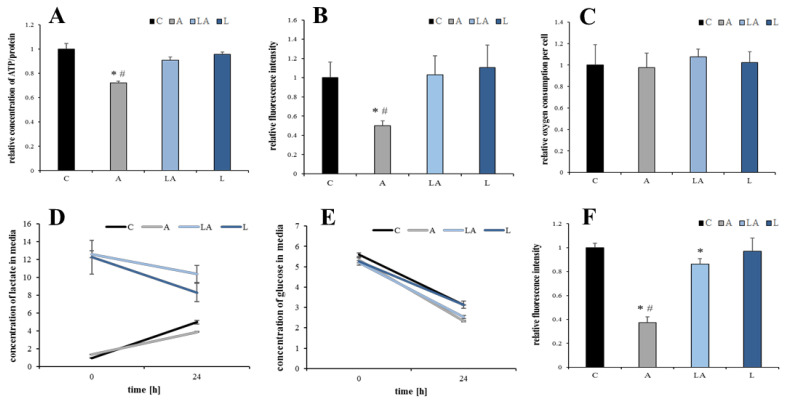
The effect of lactic acidosis on cancer cell metabolism. PaTu-8902 cells were treated with the experimental media for 72 h and (**A**) concentration of total cellular ATP, (**B**) mitochondrial membrane potential, (**C**) oxygen consumption, the changes in (**D**) lactate and (**E**) glucose levels in the culture media, and (**F**) mass of cellular phospholipids were determined. C, control; A, acidosis; LA, lactic acidosis; L, high lactate (* *p* < 0.05 compared to C, # *p* < 0.05 when comparing A and LA).

**Figure A6:**
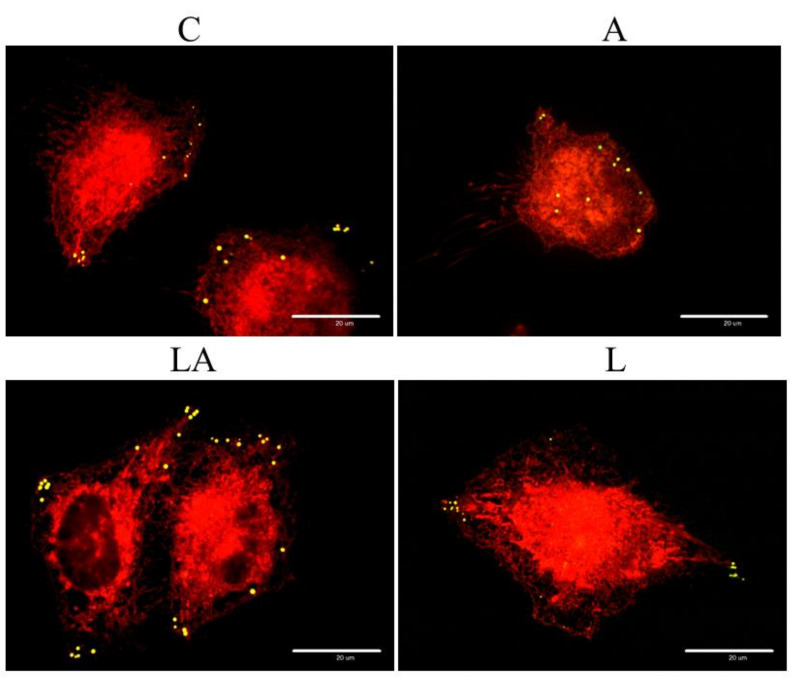
Representative images of HeLa cells stained with Nile red. HeLa cells were treated with the experimental media for 72 h, stained with Nile red, and inspected with confocal microscopy. Yellow fluorescence signal (488/525 nm) corresponds to droplets of neutral lipids and red signal (560/610 nm) corresponds to phospholipid membranes. C, control; A, acidosis; LA, lactic acidosis; L, high lactate.

Intracellular NADPH is regenerated by several cytoplasmic and mitochondrial metabolic pathways belonging to the central energetic metabolism [36] (Figure 6). Several authors described a significant metabolic rewiring of the cells under acidosis, especially increased lipid metabolism and activity of pathways known to produce NADPH [27,28,32,38,39,40]. Therefore, the effects of the experimental media on key characteristics of the cell bioenergetics were examined. The total cellular concentration of adenosine triphosphate (ATP) and the mitochondrial membrane potential dropped significantly under acidosis but not under lactic acidosis (Figure 7A,B), while the rate of oxygen consumption (Figure 7C), lactate production/utilization (Figure 7D), and glucose consumption (Figure 7E) were not significantly affected by pH of the experimental media. Importantly, acidosis in the absence of lactate significantly attenuated a red signal (560/610 nm) from Nile red staining of cells, which corresponds to the mass of phospholipid membranes (Figure 7F). The changes in green signal (488/525 nm) from Nile red corresponding to neutral lipid droplets (Figure A6) were not significant (data not shown). 

**Figure 8:**
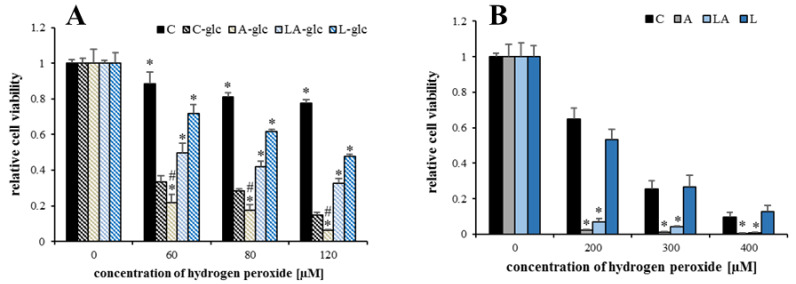
Synergic effects of lactic acidosis and metabolic manipulations. (**A**) The viability of PaTu-8902 cells treated with the experimental media lacking glucose for 72 h and challenged with the indicated concentrations of hydrogen peroxide. (**B**) The viability of PaTu-8902 cells was treated with experimental media and carbonyl cyanide-*p*-trifluoromethoxyphenylhydrazone (FCCP) for 72 h and challenged with indicated concentrations of hydrogen peroxide. Viability was measured by resazurin assay. C, control; A, acidosis; LA, lactic acidosis; L, high lactate (* *p* < 0.05 compared to C, # *p* < 0.05 when comparing A and LA).

**Figure A7:**
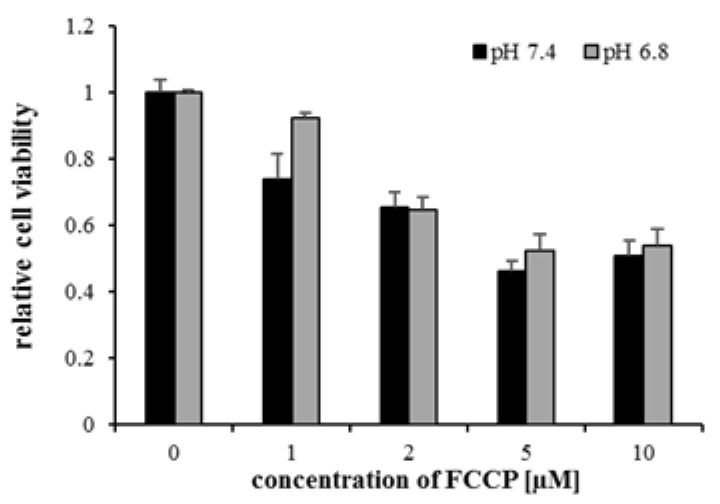
The effect of pH on cancer cell sensitivity to carbonyl cyanide-*p*-trifluoromethoxyphenylhydrazone (FCCP). Viability of PaTu-8902 cells cultured in media with different pH for 72 h and with FCCP for 24 h. Viability was measured by resazurin assay.

Based on these data, we hypothesized that the cell adaptation to acidosis is associated with enhanced cytoplasmic NADPH consumption for lipid-synthetic purposes, which requires surplus NADPH generation secured by mitochondrial metabolism of lactate. This idea was supported by performing the hydrogen peroxide challenge in the absence of glucose, the main source of cytoplasmic NADPH. The results showed that cells lacking glucose were generally much more sensitive to oxidative insult, while the effect of medium pH was minor. The presence of lactate again significantly rescued the cell viability (Figure 8A). In addition, cell treatment with a mitochondrial uncoupler carbonyl cyanide-*p*-trifluoromethoxyphenylhydrazone (FCCP), dissipating the mitochondrial membrane potential necessary for the regeneration of NADPH via nicotinamide nucleotide transhydrogenase (NNT, Figure 6), sensitized cells to oxidative stress, thus eradicating the acidosis-adapted cells independently on the presence of lactate even at the lowest concentrations of hydrogen peroxide (Figure 8B). The treatment with FCCP alone caused only a slight drop in the cell signal from the resazurin viability assay with no difference regarding the pH of the media (Figure A7).

### 2.6. Inhibition of Mitochondrial Metabolism under Acidosis 

**Figure 9:**
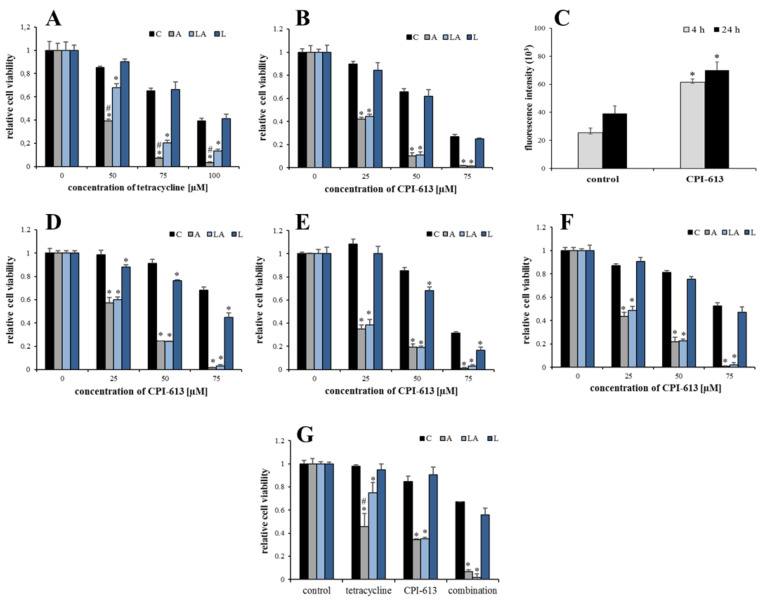
Inhibition of mitochondrial metabolism. PaTu-8902 cells were treated with the experimental media containing the indicated amount of (**A**) tetracycline or (**B**) CPI-613 for 72 h, and the viability was measured. (**C**) Oxidation of 2′,7′-dichlorodihydrofluorescein was measured as a relative fluorescence in PaTu-8902 treated with 50 µM CPI-613 for 4 and 24 h. (**D**) HeLa, (**E**) HepG2, and (**F**) HDF cells were treated with the experimental media containing the indicated amount of CPI-613 for 72 h, and the viability was measured. (**G**) PaTu-8902 cells were treated with experimental media containing 50 µM CPI-613, 50 µM tetracycline, or their combination for 2 weeks, and the viability was measured by resazurin assay. C, control; A, acidosis; LA, lactic acidosis; L, high lactate (* *p* < 0.05 compared to C, # *p* < 0.05 when comparing A and LA).

**Figure A8:**
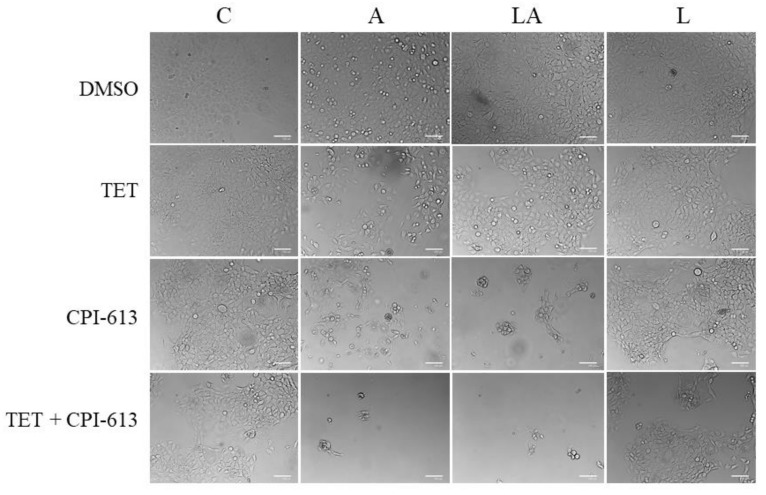
Representative images of 2D cultures treated with CPI-613 and tetracycline for 2 weeks. PaTu-8902 cells were cultured for 2 weeks in the presence of 1% dimethyl sulfoxide as a vehicle (DMSO), 50 µM tetracycline (TET), 50 µM CPI-613, or both.

**Figure A9:**
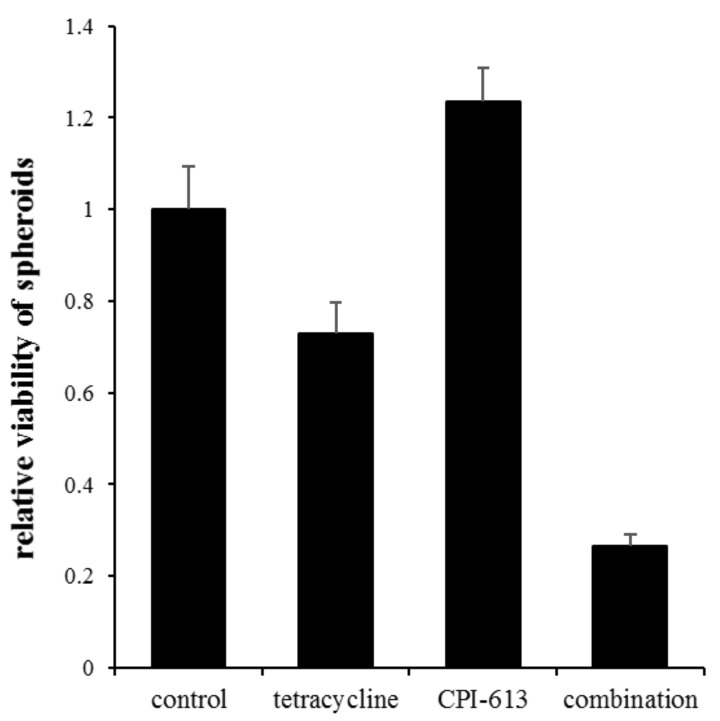
The effect of CPI-613 and tetracycline on the viability of spheroids. Viability of PaTu-8902 cells cultured in the form of spheroids for 2 weeks in the presence of 1% dimethyl sulfoxide as a vehicle (control), 50 µM tetracycline, 50 µM CPI-613, or both. Viability was measured by resazurin assay.

The observed importance of mitochondrial metabolism motivated the search for an inhibitor capable to selectively kill the cells under acidic conditions even in the presence of lactate. Tetracycline antibiotics have been previously demonstrated to suppress cancer stem cells via inhibition of mitochondrial proteosynthesis [41]. The treatment of PaTu-8902 cells with the experimental media containing tetracycline for 72 h showed the same pattern of killing as the oxidative insults (Figure 9A). Importantly, the treatment with CPI-613, an inhibitor of the pyruvate dehydrogenase complex and α-ketoglutarate dehydrogenase complex and a candidate drug against several types of cancer [www.clinicaltrials.gov [42,43,44], accessed on 15 September 2021], demonstrated a synthetic lethality with acidosis even in the presence of lactate (Figure 9B). Interestingly, the effect of CPI-613 was accompanied by the increased rate of DCF oxidation, suggesting simultaneous induction of oxidative stress enforcing its toxicity (Figure 9C). The general validity of the CPI-613 effect was confirmed also for other cell lines including noncancerous HDF (Figure 9D–F). Finally, the combination of CPI-613 with tetracycline was synergistic in a two-week experiment with PaTu-8902 cells grown in conventional 2-D cultures (Figure 9G, Figure A8) and 3-D spheroids, previously reported to be enriched with oxidative cancer cells and cancer stem cells [45] (Figure A9).

## 3. Materials and Methods

### 3.1. Chemicals

All reagents and media were purchased from Merck (Darmstadt, Germany) unless otherwise stated. Photosensitizers 5,10,15,20-tetrakis(4-isopropylphosphinatophenyl)porphyrin, herein abbreviated as TIPPP, and [Mo_6_I_8_(OCOC_4_H_8_PPh_3_)_6_]Br_4_, abbreviated as Mo cluster, were a kind gift from Dr. Kaplan Kirakci and Dr. Kamil Lang, and their synthesis and characterization were described elsewhere [46,47]. The photosensitizing properties of pheophorbide *a* were evaluated before [48]. None of these compounds showed dark toxicity at concentrations and incubation times used in this article. In general, photosensitizers, doxorubicin, tetracycline, and CPI-613 were dissolved in dimethyl sulfoxide (DMSO), and the aliquots were stored at −20 °C. Hydrogen peroxide, ascorbate, and carbonyl cyanide-4-(trifluoromethoxy)phenylhydrazone (FCCP) were dissolved in deionized water and immediately used.

### 3.2. Cell Culture

Human pancreatic adenocarcinoma PaTu-8902 cell line (DSMZ, Germany), human cervix carcinoma HeLa cell line, human hepatoblastoma Hep G2 cell line, and human dermal fibroblasts HDF (all ATCC, Manassas, VA, USA) were cultured in the Eagle’s Minimum Essential Medium (EMEM) supplemented with 0.5 mM glutamine and 5% (*v*/*v*) fetal bovine serum in 5% CO_2_ atmosphere at 37 °C. Tumor spheroids were formed in Corning Elplasia 96-well plates (Corning, NY, USA).

### 3.3. Experimental Media

Four types of experimental media were used. The control (C) medium was EMEM supplemented with 0.5 mM glutamine and 5% (*v*/*v*) fetal bovine serum. The high lactate (L) medium was enriched with 12 mM sodium lactate. Both media have a physiological pH of 7.4 in the atmosphere with 5% CO_2_. Acidosis (A) medium has adjusted pH to 6.8 with 12 mM hydrochloric acid. Lactic acidosis (LA) medium was supplemented with 12 mM lactic acid of final pH of 6.8 (Table 1).

### 3.4. Cell Viability

The cells were seeded in 96-well plates in a full cell culture medium at 20,000 cells per well. From the second day until the end of the experiment (72 h if not stated otherwise), the medium was replaced by the experimental media that were exchanged every 24 h. The viability of the cells was measured with the resazurin assay 24 h after the challenge except for X-ray-irradiated and doxorubicin-treated cells that were measured after 72 h. The X-ray irradiation was performed using X-RAD 225XL Biological irradiator (Precision X-ray, North Branford, CT, USA).

In the case of photosensitizers, cells were preincubated with the indicated concentrations of the compounds for 4 h (Mo cluster and pheophorbide *a*) or 24 h (TIPPP). The final concentration of DMSO in the medium was 1 % (*v*/*v*) including controls. The cells treated with Mo cluster were illuminated with a 460 nm LED light (Cameo, 18 mW·cm^−2^), and the cells treated with pheophorbide *a* or TIPPP were exposed to a 150 W halogen lamp equipped with a water filter (Thorlabs, 45 mW·cm^−2^) for 15 min.

### 3.5. Uptake and Intracellular Localization of the Photosensitizers

The cells were treated with experimental media and photosensitizers as described for the viability measurement, but flow cytometry measurement or confocal microscopy were performed instead of light illumination.

Uptake was determined from ten thousand cells in triplicates using a BD FACSAria III flow cytometer (excitation 405 nm, emission recorded at 655-685 nm), and the results were processed with the BD FACSDiva software (version 8, BD Biosciences, Franklin Lakes, NJ, USA).

Confocal microscopy was performed with a spinning disc microscope (Revolution xD, Andor, Oxford instruments, Abingdon, UK) operated with iQ3 software. The cells were washed and stained with LysoTracker^TM^ Green DND-26 or MitoTracker^TM^ Green (Thermo Fisher Scientific, Waltham, MA, USA). The excitation wavelengths used for monitoring the photosensitizers and lysosomes or mitochondria were 405 and 488 nm, respectively. During the confocal microscopy, the cells were maintained at 37 °C and 5% CO_2_ atmosphere.

### 3.6. Biochemical Assays

The cells were seeded and treated as described for the viability measurement, but the following assays were performed instead of challenge:

ATP concentration was determined with an ATP assay kit from Abcam (Cambridge, UK, cat.: ab113849) according to the manufacturer’s instructions.

NADPH concentrations were determined using NADP/NADPH quantification kit (cat.: MAK038) according to the manufacturer’s instructions.

Production of ROS in the cells was determined by detection of fluorescent intensity of dihydroethidium (10 mM) and 2′,7′-dichlorofluorescein diacetate (10 µM) by flow cytometry or fluorescent detector SpectraMax i3 Platform (Molecular Devices, San Jose, CA, USA). 

Relative oxygen consumption was determined by the Oxygraph+ system from Hansatech instruments (Pentney, UK). One mL of experimental media with 10^8^ cells was used for the measurement.

The total antioxidant capacity of the cell pellet was determined after resuspending in 250 µL of distilled water and 500 µL of dipyridamole (2.5 mM). Next, 80 µL of the sample was mixed with 20 µL of 2,2′-azobis(2-methylpropionamidine) dihydrochloride (25 mM) in a 96-well plate. The decrease of dipyridamole fluorescence due to oxidation was continuously monitored at 480 nm with 415 nm excitation and compared with Trolox as a standard.

### 3.7. Glutathione

Samples were extracted by 50 µL of 20 mM iodoacetamide solution in ammonium bicarbonate buffer by vortexing and incubating for 10 min at 4 °C. Then, the samples were deproteinized with 200 µL of acetonitrile/methanol mixture (1:1, *v*/*v*) and centrifuged for 10 min. The supernatants were injected onto ACQUITY UPLC BEH Amide (100 × 2.1 mm, 1.7 µm, Waters Corporation, Milford, MA, USA) column. The analysis was conducted by TSQ Quantum Access Max mass spectrometry (Thermo Fisher Scientific) in positive ionization SRM mode. The MS/MS parameters were optimized by standards through direct infusion. The selected reaction monitoring parameters are shown in Table 2. The MS detector equipped with a HESI-II probe was run under the following conditions: vaporizer temperature of 320 °C, spray voltage of +2250 V, sheath gas pressure of 34 arbitrary units (AU), the auxiliary gas pressure of 15 AU, ion sweep gas pressure of 11.2 AU, collision gas (Ar) pressure of 1.0 mTorr, and capillary temperature of 320 °C. The skimmer offset voltage was 10 V. The peaks were integrated with the Thermo Xcalibur software (Thermo Fisher Scientific).

### 3.8. Glucose and Lactate in Media

Analysis of lactate and glucose was performed by gas chromatography/mass spectrometry (Agilent Technologies, Santa Clara, CA, USA, GC7890/MS5975) in electron impact mode. Samples for the determination of lactate were extracted with a mixture of methanol, water, and chloroform (1:1:2, *v*/*v*/*v*) after the addition of the internal standard of sodium oxalate. After centrifugation at 1000× *g* for 10 min., the upper polar phase was transferred into a clean vial and lyophilized overnight. The samples were derivatized with the mixture of pyridine, *N,O*-bis (trimethyl)silylacetamide, and chlorotrimethylsilane (4:2:1, *v*/*v*/*v*). After 90 min at 65 °C, the mixture was injected onto an HP-5MS capillary column. 

Samples for the glucose determination were diluted with methanol after the addition of the internal standard D-glucose-^13^C_6_. After centrifugation at 5000× *g* for 5 min, the supernatant was transferred into a clean vial. The samples were derivatized with hydroxylamine hydrochloride in pyridine (2 mg in 150 µL of pyridine). After 30 min at 90 °C, acetic anhydride was added to the mixture. After another 30 min at 90 °C, the samples were dried out under the nitrogen and dissolved in 500 µL of chloroform before injection onto the column. The retention times and characteristic *m/z* of analyzed metabolites are attached in Table 3.

### 3.9. Statistical Analysis

Statistica software was used to assess the statistical significance of the data. Statistical differences were evaluated by one-way ANOVA followed by Duncan´s post-hoc test. Significance was set at *p* < 0.05 (* *p* < 0.05 compared to C, ^#^ *p* < 0.05 when comparing A and LA). The data were expressed as mean ± standard deviation.

## 4. Conclusions

The acidity of the tumor microenvironment has been linked to immune escape, increased invasiveness, and a worse prognosis of cancer patients. Therefore, neutralization of tumor extracellular pH is considered a promising therapeutic goal. This study presents acidosis as the Achilles’ heel of cancer. Acidosis significantly sensitized cells to hydrogen peroxide, cytotoxic doses of ascorbate, and photodynamic therapy with several photosensitizers. This effect was caused by the depletion of antioxidant capacity caused by an insufficient supply of NADPH. However, the sensitization was significantly ameliorated in the presence of respiratory substrate lactate. Inhibition of the Krebs cycle with CPI-613 resulted in selective killing of the cells adapted to acidosis even in the presence of lactate. This effect was further enhanced by tetracycline and confirmed also in tumor spheroids. In conclusion, CPI-613 and its combination with antibiotics inhibiting mitochondrial proteosynthesis could serve for selective eradication of acidosis-adapted cells, possibly improving the success rate of anticancer therapy and patient prognosis.

## Figures and Tables

**Table 1:** Experimental media.

Name	Abbreviation	Lactate [mM]	pH
Control	C	0	7.4
Acidosis	A	0	6.8
Lactic acidosis	LA	12	6.8
Lactate	L	12	7.4

**Table 2:** Selected reaction monitoring parameters for the glutathione determination.

Metabolite	Parent Ion	Product Ion	Collision Energy	Tube Lens Voltage
GSH	365.1	236.2	13 V, 20 V	93.65 V
GSSG	613.2	355.1	24 V, 40 V	122

**Table 3:** Retention times and characteristic mass/charge of analyzed metabolites.

Metabolite	*m/z*	Retention Time [min]
Oxalate (IS)	190	2.15
Lactate	219	1.63 + 4.89
Glucose	314.3	4.53
Glucose-^13^C_6_ (IS)	319.3	4.53

## Abbreviations

ATP	Adenosine triphosphate
AU	Arbitrary units
CAIX	Carbonic anhydrase IX
CAXII	Carbonic anhydrase XII
DCF	2′,7′-dichlorodihydrofluorescein
DMSO	Dimethyl sulfoxide
DNA	Deoxyribonucleic acid
EMEM	Eagle´s Minimum Essential Medium
FCCP	Carbonyl cyanide-p-trifluoromethoxyphenylhydrazone
HDF	Human dermal fibroblasts
HeLa	Human cells from cervical carcinoma
Hep G2	Human cells from hepatocarcinoma
LA	Lactic acidosis
NADPH	Nicotinamide adenine dinucleotide phosphate
NNT	Nicotinamide nucleotide transhydrogenase
PaTu-8902	Human cells from pancreatic carcinoma
PDT	Photodynamic therapy
ROS	Reactive oxygen species
TIPPP	5,10,15,20-tetrakis(4-isopropylphosphinatophenyl)porphyrin
